# Two cases of endoscopically diagnosed amebic colitis treated with paromomycin monotherapy

**DOI:** 10.1371/journal.pntd.0008013

**Published:** 2020-03-19

**Authors:** Kei Yamamoto, Yasuaki Yanagawa, Shinichi Oka, Koji Watanabe

**Affiliations:** 1 Disease Control and Prevention Center, National Center for Global Health and Medicine, Tokyo, Japan; 2 AIDS Clinical Center, National Center for Global Health and Medicine, Tokyo, Japan; 3 Department of Parasitology, National Institute of Infectious Diseases, Tokyo, Japan; University of Pennsylvania, UNITED STATES

## Presentation of cases

**Fig 1:**
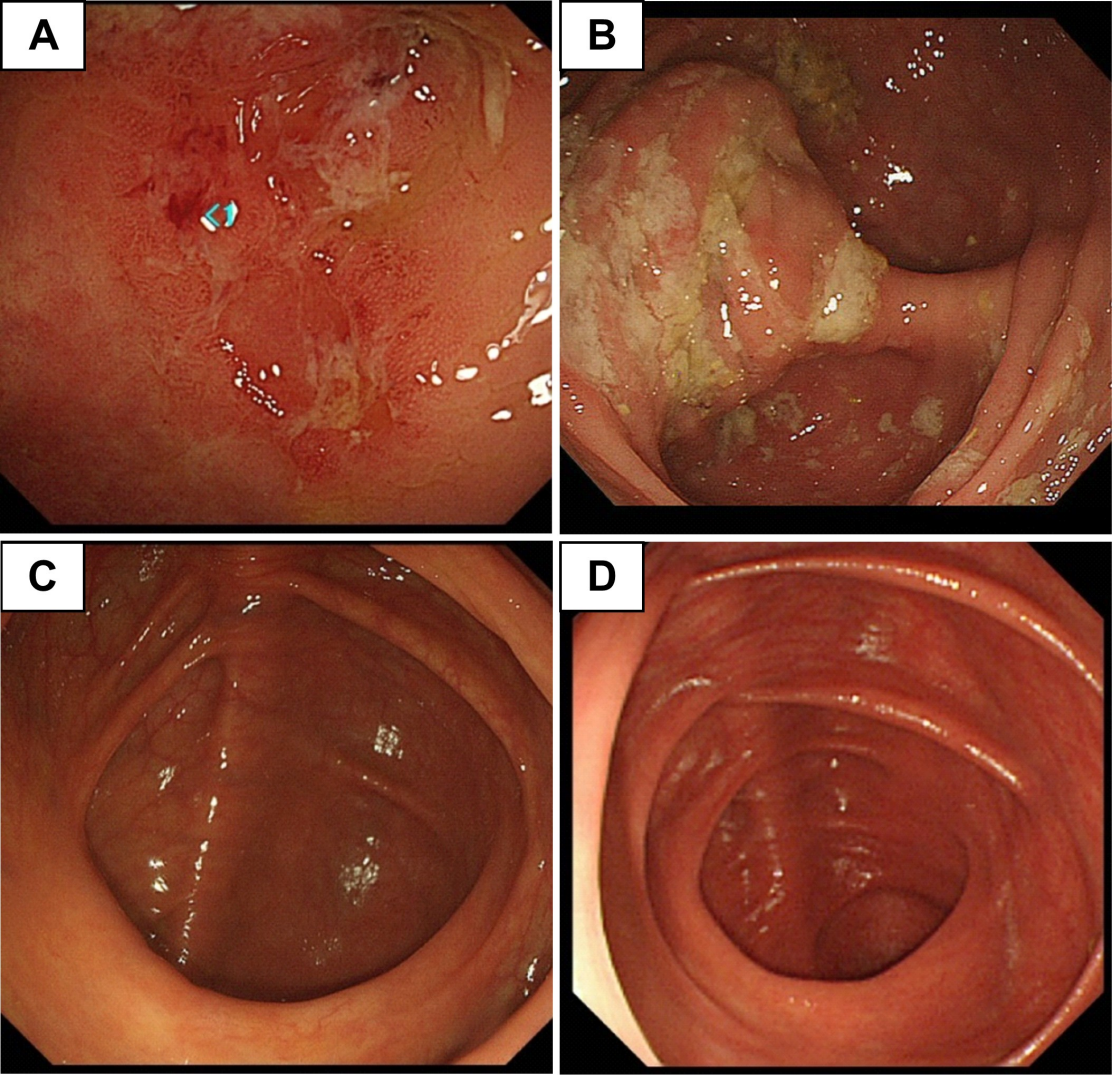
Macroscopic findings during colonoscopy. **(**A) Sporadic ulcerative lesions with edema were identified in the cecum of patient 1 before treatment. (B) Multiple ulcerative lesions with white moss were identified in patient 2 at the cecum before treatment. After completion of paromomycin monotherapy, all lesions were completely healed in (C) patient 1 and (D) patient 2.

Patient 1 who was a 43-year-old male was first referred to our institute because of an allergic reaction to metronidazole with oral mucosal erosions during his sixth treatment for amebic colitis. He had a history of 5 recurrent episodes of amebic colitis (last treatment was 3 years earlier, using metronidazole followed by paromomycin) (S1 Table). Besides oral mucosal erosions, he complained of soft or loose stools 2 to 3 times daily without abdominal pain or fever. Although we proposed admission for close observation during his treatment, he selected outpatient treatment at a nearby hospital. Three months later, the patient returned to our hospital because his wife was also diagnosed with *Entamoeba histolytica* infection. The couple operated a Japanese inn in a suburban area of Tokyo. They had no travel history to developing countries within the past 10 years. He denied extramarital sexual intercourse and oral–anal sexual contact. He did not have any past histories, except for recurrent amebiasis. There were no reported outbreaks of gastrointestinal diseases for over 10 years in the couple’s residential area. Results of a blood examination showed no particular abnormalities (Table 1). Although a direct microscopic examination was negative for any protozoa, the patient’s stool tested positive for *E*. *histolytica* with polymerase chain reaction (PCR). Total colonoscopy showed white-coated ulcerative lesions at the cecum (Fig 1A). In a pathological examination, *Entamoeba* was identified on the surface mucosa in a biopsy sample (S1 Fig). We treated the patient with a lumen-active agent (paromomycin monotherapy) because (1) he had a past history of acute oral mucosal lesions owing to metronidazole, (2) tinidazole is not approved to treat amebiasis in Japan, and (3) his symptoms of *E*. *histolytica* were mild. Negative PCR results for *E*. *histolytica* were confirmed in stool samples taken at 1, 2, and 4 months after treatment. Follow-up colonoscopy showed that lesions of the cecum were completely resolved (Fig 1C).

**Table 1:** Laboratory and endoscopic findings in the 2 cases.

	Patient 1	Patient 2
Blood tests		
White blood cell count (per μL)	5,830	6,050
Eosinophils (per μL)	140	563
C-reactive protein (mg per dL)	0.02	0.02
Antibody titer against *E*. *histolytica*, immunofluorescence assay	Negative	Positive (1:800)
Stool testing		
Fecal occult blood, immunoassay	Positive	Positive
Microscopy	Negative	Cystic form of *Entamoeba*
Antigen detection^a^	Negative	Negative
PCR for *E*. *histolytica* (tRNA STR genotype)	Positive (genotype J8)^b^	Positive (genotype J13)^b^
Endoscopic findings		
Multiple or sporadic	Sporadic	Multiple
Distribution	Cecum	Cecum ascending colon, transverse colon descending colon, sigmoid colon
Pathological findings	*Entamoeba*	Negative

^a^Tests were performed using frozen stocked samples by E. HISTOLYTICA QUIK CHEK (Techlab, Inc., Blacksburg VA, USA).

^b^Sequences of each STR genotypes are shown in S2 Fig.

PCR, polymerase chain reaction; STR, short tandem repeat; tRNA, transfer RNA

Patient 2 was a 43-year-old female and the wife of patient 1. She was referred to our institute because of *Entamoeba* infection as confirmed on microscopic examination of a stool sample. One month before diagnosis, she had a positive fecal occult blood test result in an advanced health check. Dysentery, abdominal pain, and fever were not documented at referral. She denied extramarital sexual intercourse. She had a high anti–*E*. *histolytica* antibody titer (1:800). Direct microscopy of stool samples showed the cystic form of *Entamoeba*. *E*. *histolytica* was confirmed using PCR (Table 1). Total colonoscopy showed multiple, white-coated, ulcerative lesions from the cecum to the sigmoid colon (Fig 1B). The patient was treated with paromomycin monotherapy because her symptoms were mild, and she wished to avoid the potential adverse events of metronidazole experienced by her husband. PCR for *E*. *histolytica* in stool samples collected at 1, 2, and 4 months after treatment were negative. Follow-up colonoscopy showed that all lesions had completely resolved (Fig 1D).

We informed both patients about risk behavior for acquiring *E*. *histolytica*, such as oral–anal sexual contact or food and waterborne infections in poor sanitary settings. More than 2 years after treatment, neither patient has experienced recurrence of invasive *E*. *histolytica* infection.

## Discussion

### Epidemiology of sexually transmitted *E*. *histolytica* infection

Amebiasis is transmitted by oral ingestion of the transmissible cystic form of *E*. *histolytica* in human stool. This transmission occurs by ingestion of fecally contaminated food and water, mainly in developing countries. The pathogen can also be transmitted directly from human to human, with clustering of *E*. *histolytica* strains due to sexual contact. [1, 2] Over the past 2 decades, amebic infection has been increasingly reported as a sexually transmitted infection (STI) in developed countries of East Asia and in Australia. [3] *E*. *histolytica* has also been recently recognized as a comorbidity among HIV-infected men or as a domestic STI in developed European countries. [4, 5] Neither of our 2 patients had a history of travel to a developing country in their lifetime. Additionally, we performed genotyping of 6 short tandem repeat (STR) loci in transfer RNA lesions of *E*. *histolytica* in stool samples from both patients. [6] Interestingly, the STR genotypes showed different patterns at all 6 loci between the 2 patients. However, both genotypes were identical to previously reported genotypes of Japanese origin (S2 Fig; J8 in patient 1 and J13 in patient 2). Therefore, we could not identify the transmission route of the patients’ infection. Additionally, because samples were not available at referral, we did not know the reason (reinfection, treatment failure by poor adherence, or drug resistance) why patient 1 repeatedly developed *E*. *histolytica* infection before referral.

### Treatment for endoscopically diagnosed amebic colitis

Noninvasive infections (described as asymptomatic intestinal colonization [7]) can be treated using a lumen-active agent, such as paromomycin [8], without a tissue-active agent. Tissue-active agents, such as metronidazole, should be administered before a lumen-active agent for invasive intestinal diseases (amebic colitis). However, in clinical settings similar to the 2 present patients, the difference between intestinal colonization and invasive colitis is often uncertain. [9–12] Our patients had minimal symptoms but showed macroscopically visible intestinal ulcers upon colonoscopy and microscopically identified inflammation caused by *E*. *histolytica* in biopsy samples. This difference is presumably because disease severity (colonization or colitis) in amebiasis is not currently determined using pathophysiological criteria but is based on patients’ symptoms. Furthermore, colonoscopy is not routinely recommended for asymptomatic cyst passers who may have ulcerative lesions in their large intestine, such as patient 2 (cystic form of Entamoeba in the stool and visible ulcers identified by colonoscopy). However, these cases are commonly treated by a lumen-active agent without a tissue-active agent. [7] These results raise the unresolved issue that monotherapy by a lumen-active agent can be inadequate treatment for asymptomatically infected patients with *E*. *histolytica* who are diagnosed by stool tests, such as microscopy or PCR. Additionally, as warranted in our cases, extra-intestinal lesions, such as liver abscess, should be ruled out before determining their treatment for all asymptomatically infected cases. However, imaging studies before treatment with a lumen-active agent are currently not recommended for these cases. [7] At a minimum, close follow-up is required when a tissue-active agent is not administered prior to a lumen-active agent. Additionally, nitroimidazole agents, especially metronidazole, often show toxicity in patients, such as drug hypersensitivity, neurological disorders, and sometimes Stevens–Johnson syndrome. [13, 14] Another problem in the clinical settings is that there are no available alternative regimens for invasive diseases for patients who are unable to tolerate a nitroimidazole agent. In our cases, we did not use tissue-active agents before paromomycin because of concern about adverse effects. Both patients achieved complete healing of ulcerative lesions after paromomycin monotherapy (Fig 1C and 1D). A study from Japan reported that 11 of 143 cases of amebiasis were treated with paromomycin monotherapy, although symptoms and outcomes of the treatment were unclear from the report. [15] Additionally, older reports published before the era of PCR differentiation of *E*. *histolytica* from nonpathogenic *Entamoeba* described the effectiveness of a single dose or 5 days of paromomycin, even for symptomatic amebic colitis. [16, 17] However, 6.3% to 44.0% of the cases experienced recurrence within 10 weeks of follow-up. Tissue-active agents are generally used for initial treatment, according to recent case reports of endoscopically diagnosed, asymptomatic amebic colitis. [9, 18] Furthermore, paromomycin is not systemically absorbed, and 100% is excreted in an unchanged form from stool. Taken together, these results suggest that the best treatment regimen for endoscopically diagnosed asymptomatic ulcers in the large intestine due to *E*. *histolytica* remains undetermined. Paromomycin monotherapy, which might be considered only for patients who are intolerant to nitroimidazole after excluding extraintestinal involvement, will not adequately treat all cases of intestinal amebiasis. Further investigations are required to determine appropriate treatment for asymptomatically infected patients with *E*. *histolytica* who are diagnosed via colonoscopy or stool tests.

## Conclusion

Successful treatment with paromomycin monotherapy due to metronidazole intolerance was microbiologically and endoscopically confirmed in 2 patients with mild symptoms of *E*. *histolytica* infection and visible colonic ulcers. This finding suggests that this treatment is an option for amebic ulcers in the setting of metronidazole intolerance if there is no evidence of intestinal and extraintestinal invasive diseases. Additionally, a future proof-of-concept study is required for the appropriate treatment choice for asymptomatically infected individuals.

**Key learning points**

- *E*. *histolytica* infection is a common STI in Japan, and it is a comorbidity among HIV-infected men or as a domestic STI in developed European countries.
- *E*. *histolytica* can cause endoscopically visible ulcers in the large intestine, even if the patient does not have any abdominal symptoms.
- Appropriate treatment for such cases (asymptomatic or mildly symptomatic patients with endoscopically visible colonic ulcers) is still undetermined.

## Consent for publication

Written informed consent was obtained from the patients for publication of details of the clinical courses.

## Supporting information

S1 Table Past treatment history of amebic colitis in patient 1. (DOCX)

S1 Fig Histopathological findings of endoscopically diagnosed amebic colitis in patient 1. (PPTX)

S2 Fig Schematic representation of STR types of each loci based on the nucleotide sequence of *E*. *histolytica* isolates from the present 2 cases. (PPTX)
