# Supplementary material for: Two cases of endoscopically diagnosed amebic colitis treated with paromomycin monotherapy
Source: PLoS Negl Trop Dis. 2020 Mar 19;14(3):e0008013. doi: 10.1371/journal.pntd.0008013 (PMC7081979; doi:10.1371/journal.pntd.0008013)
Supplement: S1 Table — (DOCX) [file pntd.0008013.s001.docx]

**S1 Table. Past treatment history of amebic colitis in patient 1.**

|  | Symptoms/abnormal findings | Medical test for *Entamoeba* spp. | Treatment | Outcome |
| --- | --- | --- | --- | --- |
| 9 years and 11 months previously | Fecal occult blood detected at a health checkup,  loose stool | Colonoscopy: multiple aphthous ulcers and erosions from the cecum to ascending colon  Pathological finding: *Entamoeba* was detected  Antibody titer: 1:400 | Metronidazole 1500 mg/day for 7 days | Microscopic test of the stool become negative 4 months after treatment |
| 9 years previously | Fecal occult blood detected at a health checkup | Colonoscopy: multiple aphthous ulcers and erosions  Microscopic test of the stool: *Entamoeba*-positive  Antibody titer: 1:200 | Metronidazole 1500 mg/day for 7 days | Not available |
| 7 years previously | Fecal occult blood detected at a health checkup | Colonoscopy: multiple aphthous ulcers and erosions  Antibody titer: 1:200 | Metronidazole 1500 mg/day for 10 days | Not available |
| 6 years and 2months previously | Persistent loose stool | Colonoscopy: multiple aphthous ulcers and erosions  Antibody titer: 1:100 | Metronidazole 1500 mg/day for 10 days | Not available |
| 3 years previously | Fecal occult blood detected at a health checkup | Colonoscopy: multiple aphthous ulcers and erosions  Antibody titer: 1:200 | Metronidazole 1500 mg/day for 10 days  Paromomycin 1500 mg/day for 7 days | Colonoscopy 6 months after treatment: slight redness of the intestinal membrane of the cecum |
