# Supplementary material for: Two cases of endoscopically diagnosed amebic colitis treated with paromomycin monotherapy
Source: PLoS Negl Trop Dis. 2020 Mar 19;14(3):e0008013. doi: 10.1371/journal.pntd.0008013 (PMC7081979; doi:10.1371/journal.pntd.0008013)
Supplement: S1 Fig — (PPTX) [file pntd.0008013.s002.pptx]

## Slide 1
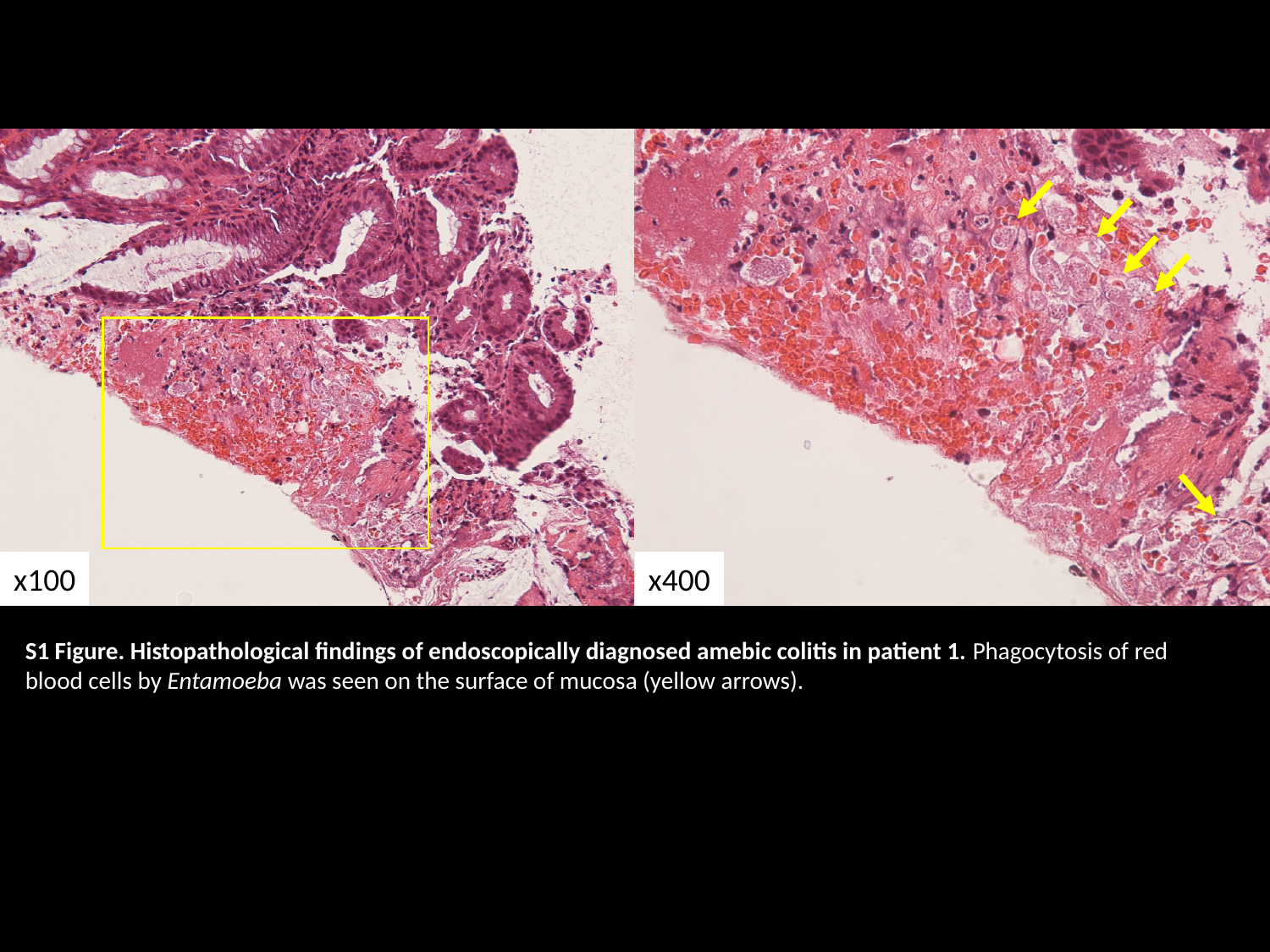

x100
x400
S1 Figure. Histopathological findings of endoscopically diagnosed amebic colitis in patient 1. Phagocytosis of red blood cells by Entamoeba was seen on the surface of mucosa (yellow arrows).
