# Supplementary material for: Two cases of endoscopically diagnosed amebic colitis treated with paromomycin monotherapy
Source: PLoS Negl Trop Dis. 2020 Mar 19;14(3):e0008013. doi: 10.1371/journal.pntd.0008013 (PMC7081979; doi:10.1371/journal.pntd.0008013)
Supplement: S2 Fig — (PPTX) [file pntd.0008013.s003.pptx]

## Slide 1
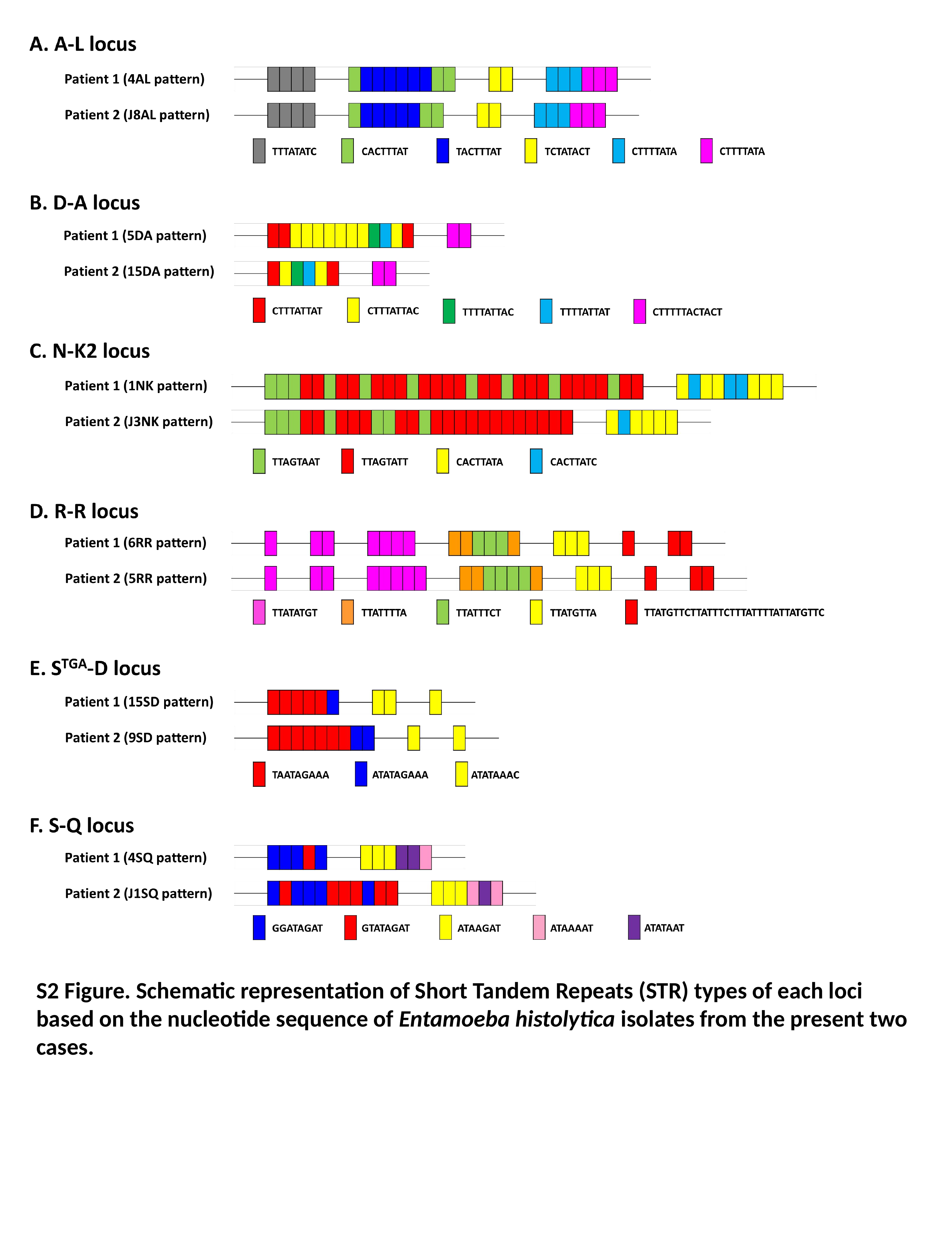

S2 Figure. Schematic representation of Short Tandem Repeats (STR) types of each loci based on the nucleotide sequence of Entamoeba histolytica isolates from the present two cases.
